# A Suggested Model for the Vulnerable Phase of Heart Failure: Assessment of Risk Factors, Multidisciplinary Monitoring, Cardiac Rehabilitation, and Addressing the Social Determinants of Health

**DOI:** 10.7759/cureus.35602

**Published:** 2023-02-28

**Authors:** Joseph Phan, Crystal Barroca, Joel Fernandez

**Affiliations:** 1 Department of Internal Medicine, Nova Southeastern University Dr. Kiran C. Patel College of Osteopathic Medicine, Clearwater, USA; 2 Division of Cardiovascular Sciences, Department of Internal Medicine, University of South Florida (USF) Health Morsani College of Medicine, Tampa, USA

**Keywords:** cardiac resynchronization therapy, hospital mortality, disease management programs, social determinants of health, remote patient monitoring, cardiac rehabilitation, heart failure, vulnerable phase

## Abstract

The vulnerable phase (VP) of heart failure (HF) is 30 to 90 days after hospital discharge and is associated with increased rehospitalization and mortality rates. The pathophysiological mechanism that drives the VP is due to the progressive increase in left ventricular filling pressure, which can cause hemodynamic congestion and long-term multiorgan injury. Our team analyzed English-written, peer-reviewed research through PubMed from 2018 to 2022, to gather current information on the VP and generate a multipronged approach toward the assessment and intervention of patients with posthospitalization HF. It is our opinion that a structured approach using remote vital monitoring and risk-stratifying tools will be best to identify patients at risk for decompensatory HF during the VP. Medical management can then be targeted toward these high-risk patients by using an organized multidisciplinary team and a disease management program, which includes remote patient-monitoring systems, addressing social determinants of health, and cardiac rehabilitation, to improve rehospitalization and mortality rates.

## Editorial

Introduction

The vulnerable phase (VP) of heart failure (HF) is 30 to 90 days after hospital discharge and is associated with a rehospitalization and mortality rate of 30% and 10%, respectively [1]. The pathophysiological mechanism that drives the VP is due to the progressive increase in left ventricular filling pressure driving hemodynamic congestion and long-term multiorgan injury [1,2]. Inability to adequately decrease congestion during the VP results in an increased risk for adverse events and death [1,2]. However, it is unknown how to assess a patient’s heightened risk during the VP and properly manage the condition using a multidisciplinary team. Appropriate timing of the medical team to initiate aggressive guideline-directed medical therapy (GDMT) during the VP is important to reduce the risk of death and rehospitalization [1]. Although some patients may have adequate cardiac care with routine advisement and monitoring, other patients with lower social, economic, and cultural capital often have difficulties in arranging outpatient follow-up, a lack of resources to pay for medications, or a lack of medical education [3,4]. Therefore, in this editorial, we suggest a model where the disease management program identifies those with an elevated risk for cardiac decompensation during the VP by using risk-stratifying tools. The disease management program will then administer care management protocols, remote patient monitoring (RPM) systems, and cardiac rehabilitation (CR) to improve rehospitalization and mortality rates not only in patients with adequate care but also specifically in those who have social determinants of health (SDOH) that limit their ability to be aggressively monitored.

Disease management programs

**Figure 1:**
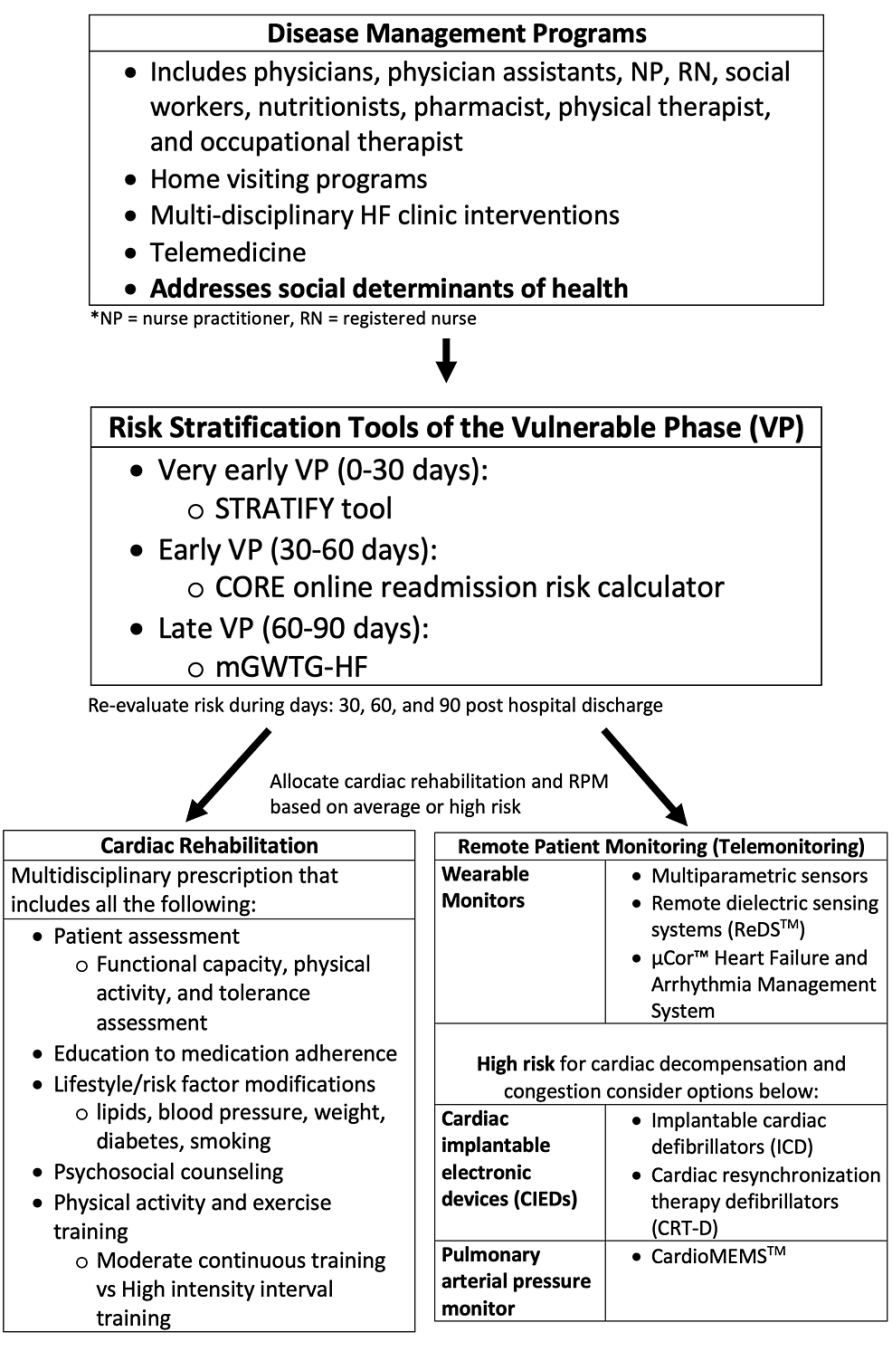
Multimodal approach in addressing the VP of acute heart failure during posthospitalization discharge. Disease management programs that include physicians, physician assistants, nurse practitioners, registered nurses, social workers, nutritionists, pharmacists, physical therapists, and occupational therapists categorize patients based on risk using risk stratification tools and provide early CR and RPM. The disease management program during the initial assessment will also consider the social determinants of health in risk stratification. Patients' risk is reassessed on posthospitalization days 30, 60, and 90 to adjust the distribution of correct resources (such as invasive RPM and more frequent CR) during the VP. Dr. Joel Fernandez developed the idea for this treatment algorithm. Joseph Phan created the computerized sketch of the figure. Inspiration for this model is derived from [1,4,5,9-11,14]. VP, vulnerable phase; ANP, atrial natriuretic peptide; BUN, blood urea nitrogen; STRATIFY, Improving Heart Risk Stratification in the ED; CORE, Center for Outcome Research and Evaluation; mGTWG-HF, modified Get With the Guidelines-Heart Failure; RPM, remote patient monitoring

The goal of a structured HF disease management program is to reduce the 30-day readmission rates for patients with HF through self-care education, utilizing the teach-back method, medication reconciliation, multidisciplinary consultation, telephone follow-up within 48 to 72 hours of discharge, follow-up visits within 7 to 10 days of discharge, and identifying the SDOH [3]. The healthcare team, which consists of physicians, physician assistants, nurse practitioners, registered nurses, social workers, nutritionists, pharmacists, physical therapists, and occupational therapists, modifies the clinical protocol based on risk and identifies referrals for advanced therapies and interventional chronic HF (CHF) [3]. The team also synthesizes information (medical history, examinations, and laboratory tests) from monitored data (RPM), implantable cardioverter defibrillator (ICD) data, diagnostic tests (echocardiography, catheterization, MRI, nuclear imaging, stress testing, and six-minute walk test), and cardiac habitation reports (Figure 1) [3,5]. A randomized controlled trial showed that this type of collaborative management, in addition to telemonitoring, decreased rehospitalization rates, all-cause mortality, and improved psychosocial well-being [5]. In addition, it is customary for the HF specialist clinician (MD, DO, PA, and NP) to coordinate tests and referrals and assess comorbidities (obstructive sleep apnea, chronic kidney disease, and diabetes mellitus) for the patients [3,5]. As a result, understanding the structured HF management program and the role of each member of the healthcare team is essential for providing adequate patient care during the VP.

Risk factors

The first important aspect to address during the VP of HF is categorizing patients based on their risk for rehospitalization and one-year mortality using applicable cardiac failure risk scores. We chose the most comprehensive risk stratification tools, which combine multiple comorbidities and laboratory results, to generate a risk score specific to very early (0-30 days) - Improving HF Risk Stratification in the ED (STRATIFY) tool; early (30-60 days) - Center for Outcome Research and Evaluation (CORE) online readmission risk calculator; and late (60-90 days) VP of HF - modified Get With the Guidelines-HF (mGWTG‐HF) scores (Table 1; Figure 1) [1,6-8]. The STRATIFY tool, CORE online readmission risk calculator, and GWTG-HF score will take into account the patients’ in-hospital clinical condition and risk of decompensation before discharge using clinical signs, symptoms, and laboratory biomarkers (Table 1) [1,6-8].

**Table 1:** Elements of the risk stratification tools used for the vulnerable phase of heart failure. ^*^Added to the Get With the Guidelines-Heart Failure (GWTG-HF) score to generate the mGWTG-HF score. COPD, chronic obstructive pulmonary disease; ED, emergency department; BNP, brain natriuretic peptide; ACE-I, angiotensin-converting enzyme inhibitors; SaO2, oxygen saturation; GFR, glomerular filtration rate; MI, myocardial infarction; LVEF %, left ventricular ejection fraction percentage; PCI, percutaneous coronary intervention; NYHA, New York Heart Association; STRATIFY, Improving Heart Failure Risk Stratification in the ED; CORE, Center for Outcome Research and Evaluation; mGWTG-HF, modified Get With the Guidelines-Heart Failure

STRATIFY tool [8]	CORE online readmission risk calculator [7]	mGWTG‐HF [6]
Age	Age	Age
Body mass index	Female	Systolic blood pressure
Plasma BNP	Body mass index	Blood urea nitrogen
Blood urea nitrogen	History of heart failure	Heart rate
Dialysis	Previous vascular disease	Sodium
Diastolic blood pressure	Chronic lung disease	COPD
Sodium	Diabetes mellitus	Race
Supplemental oxygen	GFR	NYHA functional class*
Outpatient ACE-I	Dialysis	Anemia*
Respiratory rate	Current heart failure	LVEF %*
SaO2	Symptoms of MI on admission	Plasma BNP*
Troponin	LVEF %	
	PCI status	

We propose to divide patients into average or high risk based on the established scoring guidelines of the risk-stratifying tools [1]. For the STRATIFY tool, based on the number of increasing deaths in their cohort study, average and high risks correlate to a 3% to 5% risk of events and a 5% to 10% risk of events, respectively [8]. For the CORE online readmission risk calculator, average and high risks correlate to 8% to 18.5% and >18.5% risk for 30-day readmission, respectively [7]. Finally, for the mGWTG score, based on the all-cause death and cardiac events, average and high risks correlate to GWTG-HF scores of 36-41 and 42-67, respectively, as well as the clinical judgment of the New York Heart Association (NYHA) class, presence of anemia, left ventricular ejection fraction percentage (LVEF %), and plasma brain natriuretic peptide (BNP) [6]. Patients should be deemed high risk if they have NYHA class III and class IV, the presence of anemia, plasma BNP of approximately 418.2 pg/mL or higher, and LVEF % of 48.1 ± 16.7 or lower [6].

Patients deemed to be at high risk during the VP will have a higher resource allocation of RPM (cardiac implantable electronic devices, CardioMEMS^TM^, Abbott Inc., Atlanta, GA, USA), frequency of home visits, home health, CR, and interventions addressing SDOH. Using the appropriate risk-stratification tools, patient risk during the VP of cardiac failure should be measured at monthly intervals for a total of 90 days (Figure 1). This is because as interventions are applied, the risk of mortality and hospitalizations often decrease [1,9]. One such example is shown in the CHAMPION trial (CardioMEMS^TM^ Heart Sensor Allows Monitoring of Pressure to Improve Outcomes in New York Heart Association Class III Heart Failure Patients), where if a pulmonary arterial pressure monitor, CardioMEMS^TM^, was used to guide therapy, HF and respiratory hospitalizations were reduced by 41% and 62%, respectively, validating the claim to readdress patient risk during the VP and adjusting risk resource allocation as necessary [1,9].

Remote patient monitoring

The use of RPM is important as a screener during the disease management program. Its use during the VP of cardiac failure is to detect decompensation risk, which correlates with early detection of cardiac congestion [1]. In addition to the ability of RPM to detect cardiac congestion, it may also schedule visits based on its monitoring results for clinic-based point-of-care testing (BNP and basic metabolic panel) and administration of intravenous (IV) diuretics [1].

RPM should be based on risk during the VP, as discussed earlier. Average-risk patients may be placed on noninvasive RPM technology such as wearable monitors that monitor the heart rate, blood pressure, activity, lung water content, and arrhythmias [1,10,11]. The LINK-HF study (Multisensor Noninvasive Remote Monitoring for Prediction of Heart Failure Exacerbation), which analyzed the accuracy of wearable multiparametric sensors, had a sensitivity between 76% and 88% and a specificity of 87% to detect HF hospitalization [10]. Furthermore, a meta-analysis showed that remote dielectric sensing systems (ReDS^TM^, Sensible Medical Innovations Ltd., Netanya, Israel) significantly lower the odds of hospital readmission rates [11]. Although studies are limited, a novel noninvasive device, known as µCor^TM^ HF (ZOLL Manufacturing Corporation, Pittsburgh, PA, USA) and Arrhythmia Management System, uses a radiofrequency-based thoracic fluid index (TFI) to record the risk of pulmonary edema [12]. High-risk patients will more likely benefit from invasive RPM such as ICDs, cardiac resynchronization therapy defibrillators (CRT-Ds), and pulmonary arterial pressure monitor (CardioMEMS^TM^), which have strong evidence, based on OptiLink HF (Optimization of Heart Failure Management Using Medtronic OptiVol Fluid Status Monitoring and CareLink Network) and CHAMPION trial to reduce hospitalization rates [1,9,13].

Cardiac rehabilitation

The correct prescription of CR includes a comprehensive approach that addresses nutritional counseling; lifestyle modification; risk factor management for lipids, blood pressure, weight, diabetes, and smoking; psychosocial interventions; and physical activity counseling and exercise training [14]. The physiological benefits for secondary prevention from CR stem from improvements in cardiac output, autonomic balance, endothelial function, muscle fiber composition, and respiratory muscle strength, in addition to decreasing inflammation, insulin resistance, and levels of N-terminal pro-B-type natriuretic peptide [14]. The benefits of CR are that it improves all-cause mortality over the long term (12 months follow-up), reduces overall hospital admissions in the short term (up to one year of follow-up), and HF-specific hospitalization [15].

It is important to tailor the specific mode of CR, as each mode differs in its ability to influence exercise capacity, peak oxygen uptake, health-related quality of life, HF-related hospitalization, and mortality risk [14]. The different types of modes of CR include center-based (CB-CR), home-based (HB-CR), technology-enabled (TE-CR), and hybrid [16]. CB-CR and HB-CR are physical rehabilitation that takes place either at an HF clinic or at home, respectively [14,16]. TE-CR involves telephone, telemedicine, and internet-based models of aerobic and strength training coupled with wearable technologies and implanted devices to monitor physical activity [14,16]. Unlike CB-CR and HB-CR, TE-CR facilitates patient adherence by providing flexibility in terms of having to travel, in addition to increasing participation and retention [14].

We suggest a mixture of TE-CR and CB-CR in a 1:1 ratio for those at moderate risk as it may increase its utilization by HF teams and its adherence by patients [14]. For patients who are at high risk, CB-CR will have to be more frequently utilized as it is currently the only validated method to improve rehospitalization and mortality rates based on a recent meta-analysis [15,16]. Although future studies need to determine the frequency of CB-CR used in high-risk patients, TE-CR should still be implemented as it may increase adherence to the CR program [14,16]. With enhanced physician endorsement of a hybrid program coupling multidisciplinary monitoring (using MD, advanced registered nurse practitioners, pharmacists, and physical therapists), CB-CR, TE-CR, RPM, and traditional facility-based programs, adherence to CR may become increasingly higher.

Social determinants of health

The economic, social, environmental, and psychosocial factors that influence health, also known as the SDOH, have a significant impact on cardiovascular health, particularly during the vulnerable phase [4]. The healthcare team screens and intervenes in the gaps identified for the SDOH. When assessing a patient, it is critical to consider their employment, economic status, education, and neighborhood because low-socioeconomic status has been associated with a higher risk of mortality and HF hospital readmission [4,17,18]. Additionally, physical and emotional stress can have an impact on recovery, which was shown by a regression analysis demonstrating the correlation between depression and decrease exercise capacity, measured by the six-minute walk test [4,19]. Moreover, chronic stress can promote a proinflammatory state, which plays a role in major adverse cardiac events [4].

Access to healthcare and quality education are other SDOHs to consider [4]. Although a cohort study has shown no association between health insurance status and the severity of HF, insurance coverage is important to providing access to quality healthcare and decreasing the debt burden [18]. Therefore, the disease management program will need to address healthcare access measured by the insurance status of the individual when prescribing, referring, and planning CR, RPM, and inpatient treatment [4,18]. Additionally, the lack of quality education must be recognized as it has been associated with higher odds of having severe HF (NYHA class III/IV) [18]. Therefore, the use of a translator (for those who have a language barrier), simpler words, hand gestures, and images should be utilized [4,18]. All aspects of the SDOH that influence patients' health during the VP should be addressed by the disease management program with referrals for counseling from social workers and nutrition specialists [4,17,19].

Conclusions

It is our opinion that the use of an organized multidisciplinary team to categorize risk in patients during the VP of cardiac failure to tailor management such as RPM and CR and address SDOH will holistically improve hospitalization rates and decrease mortality in HF patients. Patients with a high risk for cardiac decompensation will receive more invasive RPM, early CR, and more frequent monitoring by the disease management team. Ongoing reassessment of cardiac decompensatory risk on posthospitalization days 30, 60, and 90 will allow for the proper allocation of resources to patients during the VP. Our model also integrates the SDOH because economic, social, environmental, and psychosocial factors further contribute to the compensatory decline of patients during the VP of HF. Further studies should be focused on generating a standardized guideline to identify and provide a comprehensive management plan for patients during the VP.

## References

[1] Gracia E, Singh P, Collins S, Chioncel O, Pang P, Butler J (2018). The vulnerable phase of heart failure. Am J Ther.

[2] Fudim M, Parikh KS, Dunning A (2018). Relation of volume overload to clinical outcomes in acute heart failure (from ASCEND-HF). Am J Cardiol.

[3] Charais C, Bowers M, Do OO, Smallheer B (2020). Implementation of a disease management program in adult patients with heart failure. Prof Case Manag.

[4] Powell-Wiley TM, Baumer Y, Baah FO (2022). Social determinants of cardiovascular disease. Circ Res.

[5] Mizukawa M, Moriyama M, Yamamoto H (2019). Nurse-led collaborative management using telemonitoring improves quality of life and prevention of rehospitalization in patients with heart failure. Int Heart J.

[6] Suzuki S, Yoshihisa A, Sato Y (2018). Clinical significance of get with the guidelines-heart failure risk score in patients with chronic heart failure after hospitalization. J Am Heart Assoc.

[7] Minges KE, Herrin J, Fiorilli PN, Curtis JP (2017). Development and validation of a simple risk score to predict 30-day readmission after percutaneous coronary intervention in a cohort of medicare patients. Catheter Cardiovasc Interv.

[8] Collins SP, Jenkins CA, Harrell FE Jr (2015). Identification of emergency department patients with acute heart failure at low risk for 30-day adverse events: the STRATIFY decision tool. JACC Heart Fail.

[9] Shavelle DM, Desai AS, Abraham WT (2020). Lower rates of heart failure and all-cause hospitalizations during pulmonary artery pressure-guided therapy for ambulatory heart failure: one-year outcomes from the CardioMEMS post-approval study. Circ Heart Fail.

[10] Stehlik J, Schmalfuss C, Bozkurt B (2020). Continuous wearable monitoring analytics predict heart failure hospitalization: the LINK-HF multicenter study. Circ Heart Fail.

[11] Sattar Y, Zghouzi M, Suleiman AM (2021). Efficacy of remote dielectric sensing (ReDS) in the prevention of heart failure rehospitalizations: a meta-analysis. J Community Hosp Intern Med Perspect.

[12] Connaire JJ, Sundermann ML, Perumal R, Herzog CA (2020). A novel radiofrequency device to monitor changes in pulmonary fluid in dialysis patients. Med Devices (Auckl).

[13] Liu P, Xing L (2022). Effect of ICD/CRT-D implantation on adverse events and readmission rate in patients with chronic heart failure (CHF). Comput Math Methods Med.

[14] Bozkurt B, Fonarow GC, Goldberg LR (2021). Cardiac rehabilitation for patients with heart failure: JACC expert panel. J Am Coll Cardiol.

[15] Long L, Mordi IR, Bridges C (2019). Exercise-based cardiac rehabilitation for adults with heart failure. Cochrane Database Syst Rev.

[16] Tegegne TK, Rawstorn JC, Nourse RA, Kibret KT, Ahmed KY, Maddison R (2022). Effects of exercise-based cardiac rehabilitation delivery modes on exercise capacity and health-related quality of life in heart failure: a systematic review and network meta-analysis. Open Heart.

[17] Mathews L, Ding N, Mok Y (2022). Impact of socioeconomic status on mortality and readmission in patients with heart failure with reduced ejection fraction: the ARIC study. J Am Heart Assoc.

[18] Wajanga BM, Kim CY, Peck RN, Bartlett J, Mabula D, Juma A, Muiruri C (2022). Is lack of health insurance a predictor of worsening of heart failure among adult patients attending referral hospitals in Northwestern Tanzania?. PLoS One.

[19] Chialà O, Vellone E, Klompstra L, Ortali GA, Strömberg A, Jaarsma T (2018). Relationships between exercise capacity and anxiety, depression, and cognition in patients with heart failure. Heart Lung.

